# Ex Vivo Human Skin is not a Barrier for Cyclic Siloxanes (Cyclic Silicones): Evidence of Diffusion, Bioaccumulation, and Risk of Dermal Absorption Using a New Validated GC-FID Procedure

**DOI:** 10.3390/pharmaceutics12060586

**Published:** 2020-06-24

**Authors:** Dominika Krenczkowska, Krystyna Mojsiewicz-Pieńkowska, Bartosz Wielgomas, Dagmara Bazar, Zbigniew Jankowski

**Affiliations:** 1 Department of Physical Chemistry, Faculty of Pharmacy, Medical University of Gdańsk, Al. J. Gen. Hallera 107, 80-416 Gdańsk, Poland; d.krenczkowska@gumed.edu.pl (D.K.); dagmara-b@gumed.edu.pl (D.B.); 2 Department of Toxicology, Faculty of Pharmacy, Medical University of Gdańsk, Al. J. Gen. Hallera 107, 80-416 Gdańsk, Poland; bartosz.wielgomas@gumed.edu.pl; 3 Department of Forensic Medicine, Faculty of Medicine, Medical University of Gdańsk, ul. Dębowa 23, 80-204 Gdańsk, Poland; zbigniew.jankowski@gumed.edu.pl

**Keywords:** cyclic siloxanes, silicones, human skin diffusion, percutaneous absorption, transdermal diffusion Franz cell, gas chromatography-flame ionization detector (GC-FID)

## Abstract

Cyclic methylsiloxanes D4, D5, D6 (also called cyclic silicones) are widely used in various dermatological products and cosmetics, both for children and adults. As a result of their unique physicochemical properties, the production of cyclic methylsiloxanes has greatly increased over the last few years, which has resulted in increased exposure to mankind. The validated quantitative for gas chromatography-flame ionization detector (GC-FID) analysis with using the transdermal diffusion system with vertical Franz cells demonstrated that ex vivo human skin is not a barrier to cyclic siloxanes. D4, D5, and D6 have a specific affinity to stratum corneum (SC) (especially D6), and can even diffuse into the deeper layers of the skin (epidermis (E) and dermis (D)), or into the receptor fluid as well. An important achievement of this work was the observation of the characteristic ratio partitioning D4, D5, and D6 in skin layers and receptor fluid (RF). The studies have shown that, in order to thoroughly understand the mechanism, it is important to determine not only the differences in the amounts of cumulated doses in total in all skin layers and receptor fluid, but also the mutual ratios of analyte concentrations existing between matrices. For example, in the case of the stratum corneum, the cumulative doses of D4, D5, and D6 were 27.5, 63.9, and 67.2 µg/cm^2^/24 h, respectively, and in the epidermis, they were 6.9, 29.9, and 10.7 µg/cm^2^/24 h, respectively, which confirmed the highest affinity of D6 to stratum corneum as the amount diffused into the epidermis was 2.8 times smaller compared to D5. The calculated epidermis-to-stratum corneum ratios of analyte concentrations also confirm this. The largest ratio was identified for D5 (E/SC = 47), followed by D4 (E/SC = 25), and finally by D6 (E/SC = 16). The analysis of the next stage of diffusion from epidermis to dermis revealed that in dermis the highest cumulative dose was observed for D5 (13.9 µg/cm^2^/24 h), while the doses of D4 and D6 were similar (5.1 and 5.3 µg/cm^2^/24 h). Considering the concentration gradient, it can be concluded that the diffusion of D5 and D6 occurs at a similar level, while D4 diffuses at a much higher level. These observations were also confirmed by the dermis-to-epidermis concentration ratios. The final stage of diffusion from dermis to the receptor fluid indicated that D4 was able to permeate easily, while D5 exhibited a difficult diffusion and the diffusion of D6 was limited. The receptor fluid-to-dermis concentration ratios (RF/D) were calculated for D4, D5, and D6: 80, 53, and 17, respectively. Our results also revealed the increased risk of D4 and D5 absorption into the blood and lymphatic systems, whereas D6 demonstrated the lowest risk. Therefore, we can argue that, among the three tested compounds, D6 is the safest one that can be used in dermatological, cosmetic, and personal care products. This study demonstrates that the stratum corneum, epidermis, and dermis can be also considered reservoirs of cyclic methylsiloxanes. Therefore, these compounds can demonstrate potential long-term bioaccumulation, and can be absorbed to the bloodstream in a long-term and uncontrolled process.

## 1. Introduction

Cyclic volatile methylsiloxanes (cVMS), including D4 (octamethylcyclotetrasiloxane), D5 (decamethylcyclopentasiloxane), and D6 (dodecamethylcyclohexasiloxane, belong to one of the most popular chemical groups called silicones [1,2]. They have been widely used in dermatological, cosmetic, personal care, and household products. They are used in the production of silicone polymers, and have a range of industrial applications, including pharmacy and medicine [3,4,5,6]. For many years, their use was considered safe for humans [3,7]; however, in recent years, it has been shown that they cause environmental pollution [1,2,8,9,10,11,12,13,14,15,16]. In addition, these compounds can bioaccumulate in the living organisms [17,18,19,20], in the ecosystem with significant durability and the ability to transport to very remote areas, including the Arctic region [20,21,22]. This is mainly possible due to their physicochemical properties, such as high volatility (4.6–132 Pa at 25 °C), Henry’s law constant (1.210–4.945 kPa m^2^/mol at 25 °C), chemical stability [23], hydrophobicity (e.g., low water solubility: 5.3–56 mg/L), and high octanol-water partition coefficient (log Kow: 5.10–8.87 at 25 °C) [24,25].

The presence of cyclic siloxanes in the environment has encouraged various groups of researchers to explore the exposure of these compounds to humans (e.g., in outdoor and indoor air, workplaces, breast milk, drinking water, food, medical devices, and in cosmetic products) and their toxicological assessment. Therefore, a regulatory process has been initiated in 2008, Regulation (EC) No. 1272/2008, for the classification, labeling, and packaging of substances and mixtures. Based on this regulation, D4 has been classified in the category of Carcinogenic, Mutagenic, or Toxic for Reproduction (CMR, category 2) [26]. In addition, in 2015, the Member State Committee of the European Chemical Agency (ECHA) stated that D4 and D5 both meet the very persistent/very bioaccumulative substances (vPvB) criteria presented in Annex XIII of REACH. Furthermore, D4 is classified as category 4, which means it is toxic for the aquatic environment, and may cause long-lasting harmful effects on aquatic life. On 27 June 2018, D4, D5, and D6 were identified by ECHA’s Member State Committee as Substances of Very High Concern (SVHC) for Authorization with vPvB properties. D4 was also identified as having persistent, bioaccumulative, and toxic (PBT) properties [27,28]. Finally, in 2018, both D4 and D5 were added to ECHA’s restricted substances list, contained in Annex XVII of REACH [29]. Under Commission Regulation (EU) 2018/35, which was published on 10 January 2018, D4 and D5 can no longer be used in the production of wash-off cosmetic products at a level equal to or greater than 0.1% (by weight of either substance) after 31 January 2020 [30]. Currently, it is considered that emissions and subsequent exposure, in the case of a PBT/vPvB substance, can be considered as a proxy for unacceptable risk. The proposed restriction is not only for D4 and D5, but also for D6, because of their similarity in chemical structure, physicochemical properties, and hazard profile.

Apart from estimating environmental pollution, it is important to assess the levels and effects of human exposure to cyclic siloxanes. According to the reports published by the government and various nonprofit organizations, there is an increased risk of human exposure to cyclic siloxanes through the respiratory tract [31], but there is insufficient data regarding human exposure through the skin [7]. So far, dermal exposure of these compounds has been evaluated only based on their content in personal care products and cosmetics or blood and plasma after the application of the products. For example, the researchers analyzed 123 products (e.g., moisturizers, deodorants, body and hair washes, toilet soaps, toothpastes, and shaving products), and the presence of linear and cyclic siloxanes was confirmed in 96% of products. In conclusion, D4 and D6 were the most frequently detected compounds (85% and 78%, respectively), while D3 and D5 were detected in higher concentrations (nd–1203 µg/g; mean: 82.78 µg/g) and (nd–753.5 µg/g; mean: 43.98 µg/g) in comparison to D6 (nd–594.2 µg/g; mean: 35.84 µg/g) and D4 (nd–267.03 µg/g; mean: 25.11 µg/g) [32]. Another study showed that cosmetics and personal care products contained varying concentrations of D4, D5, and D6. Among these, the concentration of D5 was high in antiperspirants (356 mg/g) and liquid foundation (107 mg/g) [33]. According to another study, D5 was also the most predominant cVMS with the highest concentration (680 mg/g) detected in antiperspirants and baby products (150 mg/g in diaper cream) [34]. Another study verified that D4, D5, and D6 were the most detected cyclic siloxanes, with a mean concentration of 0.13 mg/g, 0.054 mg/g, and 0.016 mg/g, respectively, in hair care products [35]. Despite these studies, there is limited data available on the levels of these compounds in the human plasma of the general population, not from the occupationally exposed samples. According to Hanssen et al. [36], the plasma levels of cVMS in a pregnant and postmenopausal Norwegian woman (2007 and 2009, respectively) showed the dominant presence of D4; its concentration was the highest among the tested siloxanes (12.7 ng/mL and 2.69 ng/mL, respectively). The levels of D5 and D6 were below the limit of detection (LOD). Fromme et al. [37] presented plasma levels of siloxanes in German adults, the concentrations ranged from <0.18 to 0.73 mg/L for D4, <0.29 to 0.48 mg/L for D5, and <0.44 to 0.79 mg/L for D6.

Despite a great deal of effort laid on estimating the effect of human exposure to cVMS, studies regarding the ability of siloxanes to overcome the skin’s barrier system are scarce. Analyzing this is one of the principal stages of a reliable assessment on human exposure, and it is essential to obtain information about the mechanism of cVMS in overcoming the skin’s barrier system. So far, there are only a few studies analyzing the percutaneous diffusion of cyclic siloxanes; however, the data is insufficient. Zareba et al. [38] studied the level of siloxanes after dermal application in epidermis and dermis (in human epidermis, dermis, and adipose tissue the content was 470, 220, and 75 ng of D4, respectively); however, they did not estimate the levels in the stratum corneum or in the receptor fluid. Generally, apart from these data, the authors presented results as percent of applied dose (%), which is not sufficient to estimate an ability of absorption siloxanes through skin. According to other researchers (Jovanovic et al.) the amounts of siloxanes were quantified only in the receptor fluid (1.1 μg/cm^2^ for D4 and 0.8 μg/cm^2^ for formulated D4) and the total content in the skin (0.1 μg/cm^2^ for D5 and 0.3 μ/cm^2^ for formulated D5) [39]. The researchers proved that only 0.5% of applied D4 and 0.04% D5 were absorbed, but more than 90% was determined in the skin [39]. Apart from this, in relation to the summary, the data were presented as a percent of applied dose and not as an amount [µg/cm^2^/24 h] (in vitro percutaneous absorption—0.5% and 0.04% of applied D4 and D5, respectively, was absorbed, in in vivo studies, less than 1.0% of the applied D4 and 0.2% of applied D5 was absorbed). The authors suggest that it is necessary to take the content of siloxanes in all layers of skin (stratum corneum (SC), epidermis (E), and dermis (D)) and the receptor fluid (RF) into account for an accurate determination of penetration and permeation of siloxanes. In 2019, we published an article fulfilling this requirement [7]. In this study, we present the complete quantitative and qualitative data for cyclic siloxanes D4–D6, which we developed and validated using the gas chromatography-flame ionization detector (GC-FID) technique. This is a fast and specific test that has been thoroughly validated by our team. In addition, we explain the mechanism of how siloxanes overcome the skin’s barrier system. This study shows which cyclic siloxanes should be eliminated or used in limited quantities in dermatological products. To the best of our knowledge, this is the first study conducted on the assessment of exposure of human skin to cyclic siloxanes. The proposed methodology can be also useful in analyzing other lipophilic compounds in matrices of the human skin layers, because we solved the essential practical problem, consisting of the isolation of lipophilic substances from a complex biological matrix belonging to the human skin. There was a risk that extract with cyclic siloxane could be contaminated, e.g., by ceramides, fatty acids, cholesterol, cholesterol sulfate, and sterol esters—various proteins which are the components of skin layers [40,41].

## 2. Materials and Methods

### 2.1. Reagents

Sodium chloride (Polish Chemical Reagents, Gliwice, Poland); Igepal^®^ CA-630 (according to IUPAC octylphenoxy poly(ethyleneoxy)ethanol; C_8_H_17_–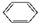
–O(CH_2_CH_2_O)_8_CHCH_2_OH; CAS Number 9002-93-1; HLB = 13) (Sigma-Aldrich, Steinheim, Germany); sodium azide (Sigma-Aldrich, Steinheim, Germany); purified water (System Elix 3, Millipore, Bedford, USA); carbon tetrachloride (CCl_4_; J.T Baker, Deventer, Holland); 1,4-dinitrobenzene (DNB); Sigma-Aldrich, Steinheim, Germany); in flame-ionization detection; ultra-high purity hydrogen, ultra-high purity nitrogen; and synthetic air (TEMIS) were used [7].

### 2.2. Tested Cyclic Siloxanes

Analytical standards of the cyclic siloxanes octamethylcyclotetrasiloxane (D4) with molecular weight MW = 297 Da, lipophilicity logP = 5.10 and solubility in H_2_O 5.6 µg/L, decamethylcyclopentasiloxane (D5) with MW = 371 Da, logP = 8.09 and solubility in H_2_O 5.1 µg/L, dodecamethylcyclohexasiloxane (D6) with MW = 445 Da, logP = 8.87 and solubility in H_2_O 1.7 µg/L, were delivered by Sigma-Aldrich, Steinheim, Germany [7,42,43,44].

### 2.3. Ex Vivo Human Skin as a Biological Sample

In this study, ex vivo human cadaver skin was used which was obtained during necropsy (in the period of 24–48 h after the time of death). Skin samples from the abdominal region with the following dimensions came from 6 males and 6 females who were in the age group of 35–50 years (the average age of females was 40 years and of males 45 years): 15–20 cm length and 2–3 cm width. The approval to use human cadaver skin for our experiments was approved by the Independent Bioethics Commission for Research at the Medical University of Gdańsk (no NKBBN/309/2013, 8 July 2013. The subcutaneous fat was removed, and the pieces were purified using 0.9% sodium chloride solution. After drying, in accordance with the guidelines provided by the Organization for Economic Cooperation and Development (OECD) and World Health Organization (WHO), the skin strips were cut into smaller strips, wrapped using aluminum foil, and stored at −20 °C until analysis.

### 2.4. Apparatus and Chromatographic Conditions

In this study, we used GC-FID equipped with autosampler (system Bruker Scion 456 GC, UK) for the analysis of cyclic siloxanes. The integrated data system was Galaxie Workstation software. The optimized parameters are presented in a previous publication of the authors [7].

### 2.5. Preparation of Calibration Standards

Stock solutions of D4, D5, and D6 at a concentration of 100 μg/mL and 10 μg/mL were prepared in CCl_4_. The following concentrations were used to prepare the calibration curve of D4, D5, and D6: 0.5, 1.0, 1.5, 2.0, 5.0, 10.0, 15.0, 25.0, 30.0, 35.0 μg/mL. These standard solutions were further diluted with an appropriate volume of CCl_4_ to prepare the working solutions. The stock and working standard solutions of siloxanes were stored at 4 °C until use. Each standard solution contained DNB as an internal standard at a concentration of 5 µg/L.

### 2.6. Preparation of Ex Vivo Human Skin and Ex Vivo Penetration and Permeation Studies

**Figure 1:**
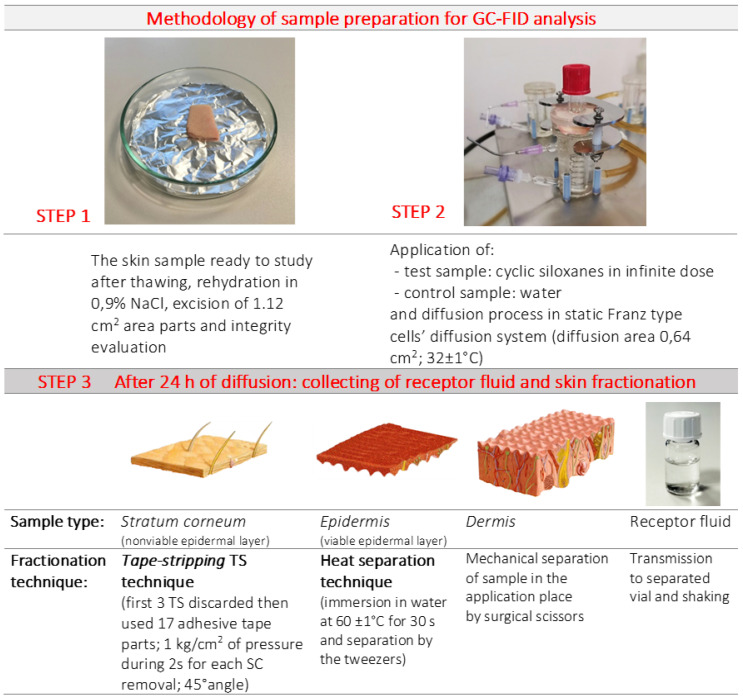
The methodology of sample preparation for gas chromatography-flame ionization detector (GC-FID) analysis.

This study was conducted in accordance with the binding guidelines for the skin absorption study, provided by the OECD and WHO [45,46,47,48]. The procedures for the ex vivo human skin preparation (Figure 1) and integrity examination were according to Krenczkowska et al. [7,44,48,49,50]. The transepidermal electrical resistance (TEER) assay was used to examine the integrity of the skin samples (determined mean 5 kΩ/cm^2^). The average full-thickness of human skin was 1.40 ± 0.2 mm.

The receptor fluid was an aqueous solution with the following composition: 2% Igepal CA-630 (as a surfactant to increase cyclic siloxanes solubility), 0.005% of sodium azide (as a preservative), and 0.9% sodium chloride. In this study, the solubility of the cyclic siloxanes in receptor fluid was about 200 µg/mL. For all siloxanes, the infinite doses were applied (100 µL, which corresponded to about 95,600 mg). The skin was exposed to cyclic siloxanes for 24 h.

### 2.7. GC-FID

In this study, GC-FID equipped with autosampler was used to analyze cyclic siloxanes. After ex vivo penetration and permeation studies [7], four types of samples were obtained and inserted into separate vials: stratum corneum layer isolated by tape-stripping (TS) technique (17 parts of adhesive tapes were collected), separated epidermis and dermis, and harvested receptor fluid. In order to extract the analytes from each sample, 990 µL of CCl_4_ (solvent) and 10 µL of 0.5 mg/mL DNB in CCl_4_ (role of the internal standard) were added, and the final volume was made up to 1 mL with the obtained solution. In the case of extraction of siloxanes from the stratum corneum samples, the procedure was altered because of the presence of adhesive tapes in vials. Therefore, we added 5 times greater volumes of CCl_4_ and DNB, and the final volume of the solution was made up to 5 mL. The final concentration of the internal standard solution was kept constant (5 µg/mL). After overnight extraction, the extracts were filtered by using a syringe filter (0.45 μm), and then put into chromatographic vials.

## 3. Validation of the Analytical Procedure and Acceptance Criteria

This is a new research topic, so it was necessary to validate the analytical method developed in this study. The proposed method was validated for the specificity, linearity, sensitivity, precision, repeatability, accuracy, quality control samples with biological matrix, matrix factor (MF), recovery (matrix effect), limit of detection (LOD), and limit of quantification (LOQ), as recommended in the United States Food and Drug Administration (FDA), European Medicines Agency (EMA), and International Conference on Harmonization (ICH) method validation guidelines [51,52,53,54]. The acceptance criteria for the validation parameters were adopted in accordance with the FDA guidelines for the validation of bioanalytical methods (Table 1) [51,52,53,54].

### 3.1. Qualitative Studies

In this study, we confirmed the specificity of the analytical method. The signal of the analyte was distinguished from the signals obtained from the various matrix (blank samples). Blank samples of the appropriate biological matrix (extracts from the isolated skin layers: stratum corneum, epidermis, dermis, as well as receptor fluid) were obtained from at least six individual sources. The specificity of the method was determined by the comparison of the chromatograms with the presence and absence of skin matrix from each layer (stratum corneum, epidermis, and dermis) and receptor fluid. The comparative analysis consisted of six different blank skin matrix extracts of each layer and receptor fluid extracts with extract samples spiked with the selected cyclic siloxanes. The analysis was performed due to the verification if, at the retention time for the siloxanes peaks, there are no peaks originated from the matrix sample, for instance from the solvent used, the internal standard IS, which was DNB, extracted the matrix of skin layers and receptor fluid. In this study, we assessed the specificity of the analytical method and obtained reliable results not only for the mean retention time (*t*_r_) of the chromatographic peaks, but also for the precision, repeatability, and intermediate precision. In order to achieve this, we measured *t*_r_ thrice for the three different concentrations of siloxanes: 1, 5, and 25 μg/mL, within three independent series of standard solutions. Thus, we verified whether the mass of the analyte and its quantity introduced into the column would affect the retention time. For each concentration cyclic siloxanes, the precision of *t*_r_ measurements was determined by calculating the relative standard deviation (RSD) values, and expressing the value in (%), also called the coefficient of variation (CV) (%). The repeatability of *t*_r_ measurements was calculated as the mean of RSD (%), obtained for the three tested concentrations. We also determined the intermediate precision based on the acquisition data of more than 4 weeks. The impact of the matrix on the precision and repeatability of *t*_r_ measurements was also conducted. The results were obtained from the recovery of cyclic siloxanes added to CCl_4_ extracts from the stratum corneum, epidermis, dermis, and receptor fluid at concentrations of 1, 5, and 25 μg/mL. We compared the results of precision and repeatability test between the samples containing CCl_4_ (standard solutions) and extracts from the layers of the skin, and the impact of the matrix on the dispersion of the retention time of D4, D5, and D6. In addition, the variability of the retention time for the standard was conducted analogously for the internal standard (DNB) added at a concentration of 5 μg/mL to all solutions containing siloxanes (reference and recovery tests). Due to the fact that this study includes biological samples for testing, it was necessary to optimize the chromatographic process to obtain adequate separation of the peaks.

### 3.2. Quantitative Analysis

For the quantitative analysis, D4, D5, and D6 working standards were prepared in a concentration range of 0.5–35 μg/mL was. To choose the right concentration range, we conducted preliminary studies. The measurement range is a validation parameter defined as the interval between the lowest and the highest concentration (along with those concentrations) that can be determined by a given method of measurement with the assumed precision, accuracy, and linearity. In this study, we prepared a calibration curve consisting of 0.5, 1.0, 1.5, 2.0, 5.0, 10.0, 15.0, 25.0, 30.0, and 35.0 μg/mL D4, D5, and D6.

#### 3.2.1. Linearity

Linearity was determined by plotting a standard curve from the peak area obtained for D4, D5, and D6 versus their corresponding concentrations. To determine the linearity, three independent calibration curves for three measurements for each concentration were conducted. In total, we obtained nine measurements for each concentration. As the dependent variable (*y*), an area ratio (A_siloxane_/A_DNB_) was assumed, where DNB was the internal standard (5 µg/mL). The concentration of D4, D5, and D6 in the samples was calculated based on the regression equation, and based on the precision, repeatability, and accuracy.

#### 3.2.2. Precision/Repeatability

Precision, which determines the closeness of agreement (degree of scatter), was expressed as a coefficient of variation (CV) (%), adopted from the RSD. Repeatability, which determines the inter-day precision, was expressed as CV (%). The experiments were performed at three different concentration levels of quality control (QC) solutions (low-, medium-, and high-quality control; LQC, MQC, and HQC, respectively). LQC, MQC, and HQC were taken at a level 1, 5, and 25 µg/mL, respectively. It should be emphasized that, due to the limited access to the biological material, the standard curve was prepared without the skin matrix; therefore, the calculated precision and repeatability did not take the matrix effect into account. For each concentration, we obtained nine results from the three independent samples and measured thrice.

#### 3.2.3. Accuracy/Trueness

The accuracy of the method was computed as the percentage of the systematic error, which is the closeness of agreement between the measured value and the true value. Similar to the determination of precision, we used three concentration levels of QC solutions: LQC, MQC, and HQC. It should be emphasized that, due to the limited access to the biological material, a significant amount of the solution necessary to performed the standard curve, these solutions were made without the matrix; therefore, the calculated accuracy did not take the matrix effect into account. The recovery test, which is a measurement of accuracy, was carried out in a separate experiment.

#### 3.2.4. QC Samples and Recovery (Extraction Efficiency)

QC samples in low (LQC), medium (MQC), and high (HQC) range of the analyte were prepared from the fractionated layers of the skin (stratum corneum, epidermis, and dermis), as well as from the receptor fluid. An appropriate volume of the solution with D4, D5, or D6 was added to the samples, in order to obtain final concentrations of 1, 5, 25 μg/mL. To obtain 1 μg/mL, we used 100 μL of 10 μg/mL stock solution; to obtain 5 μg/mL, we used 500 μL of 10 μg/mL stock solution; and to obtain 25 μg/mL, we used 250 μL of 100 μg/mL stock solution. QCs were prepared from separate stock solutions than that of the calibration standards. To each of the samples, we added DNB solution (internal standard) to obtain the concentrations of 5 μg/mL. Next, the extraction process was conducted according to the description in Section 2.7.

For the validation of the extraction process, three different concentrations (1, 5, and 25 µg/mL) of D4, D5, and D6 (dissolved in solvent-carbon tetrachloride) were added to the blank samples, which were prepared according to the procedure described in Section 2.6. The extraction recovery was determined by calculating the ratio of the amount of each siloxane extracted from the blank spiked (matrix samples) to the amount of siloxanes that were added. The recovery was verified, following the calculations from the formula, where A—peak area (n = 6):

$$\mathrm{Recovery}=\frac{\mathrm{analyte}\;\mathrm{response}\;\left(A\;\mathrm{siloxane}/A\;\mathrm{DNB}\right)\;\mathrm{present}\;\mathrm{in}\;\mathrm{blank}\;\mathrm{spiked}\;\left(\mathrm{matrix}\right)\;\mathrm{at}\;\mathrm{LQC},\;\mathrm{MQC}\;\mathrm{and}\;\mathrm{HQC}\;\mathrm{post}-\mathrm{extraction}}{\mathrm{analyte}\;\mathrm{response}\;\left(A\;\mathrm{siloxane}/A\;\mathrm{DNB}\right)\;\mathrm{present}\;\mathrm{in}\;\mathrm{blank}\;\mathrm{spiked}\;\left(\mathrm{matrix}\right)\;\mathrm{at}\;\mathrm{LQC},\;\mathrm{MQC}\;\mathrm{and}\;\mathrm{HQC}\;\mathrm{pre}-\mathrm{extraction}}\times 100\%$$

#### 3.2.5. LOD and LOQ

In this study, we also determined the LOD and the LOQ, which are important characteristics of an analytical process. To determine these parameters, one of the three recommended parameters was used, which is calculating the standard deviation of the *y*-intercept of the line “b” from nine independent calibration curves (three independent calibration curves measured thrice) and the slope “a” from the mean calibration curve, responsible for its inclination. Therefore, the minimum concentration of D4, D5, and D6 to be detected and quantified (LOD and LOQ, respectively) was calculated based on the standard deviation (σ) and on the slope (S) of the calibration curve, according to the equations (I) LOD = 3.3 σ/S and LOQ = 10 σ/S. According to the ICH guidelines, the precision and accuracy were adopted, as in the case of LLOQ (FDA, EMA, and ICH guidelines for bioanalysis), i.e., to ±20%.

#### 3.2.6. Matrix Effect and Matrix Factor

The matrix effect is a direct or an indirect alteration or interference in response to the presence of any unintended analytes or other interfering substances in the sample [56]. In this study, the MF, which expressed the matrix effect, was verified, following calculations from the formula, where A is the peak area (n = 6):

$$MF=\frac{\mathrm{analyte}\;\mathrm{response}\;\left(A\;\mathrm{siloxane}/A\;\mathrm{DNB}\right)\mathrm{from}\;\mathrm{blank}\;\mathrm{spiked}\;\left(\mathrm{matrix}\right)\mathrm{at}\;\mathrm{LQC},\;\mathrm{MQC}\;\mathrm{and}\;\mathrm{HQC}\;\mathrm{post}-\mathrm{extraction}}{\mathrm{analyte}\;\mathrm{response}\;\left(A\;\mathrm{sloxane}/A\;\mathrm{DNB}\right)\;\mathrm{from}\;\mathrm{spiked}\;\mathrm{solvent}\;\mathrm{at}\;\mathrm{LQC},\;\mathrm{MQC}\;\mathrm{and}\;\mathrm{HQC}\;\mathrm{used}\;\mathrm{for}\;\mathrm{extraction}}$$

Enriched samples containing the matrix were prepared according to the description provided in Section 2.7. It was assumed [56] that if the value of MF is close to 1, then the matrix effect is negligible. If it is significantly less than 1, it would indicate a suppressive effect (weakening of response). However, a value greater than 1 would mean an enhancement of the analyte’s response.

## 4. Results and Discussion

This study is a continuation of our previous study [7], which outlined the mechanism of penetration and permeation od cyclic siloxanes through the dermal layers. In this study, we focused on the verification of the possibility of D4, D5, and D6 to overcome the skin’s barrier system and bioaccumulate in the stratum corneum, epidermis, dermis, as well as in the receptor fluid, and in addition, we sought to understand the mechanism of diffusion of cyclic siloxanes through the skin. Apart from this, we aimed to develop and validate the procedure for the quantification of low-molecular weight cyclic siloxanes, namely, D4, D5, and D6 in the fractionated layers of ex vivo human skin, as well as in the receptor fluid, using GC-FID and the system of transdermal diffusion with vertical Franz cells.

### 4.1. Optimization of the Method and Extraction of the Dermal Layers

The optimization of analytical procedure was necessary because of the complexity of skin matrix. The use of hydrogen as the carrier gas, due to its most advantageous separation power, enabled satisfactory separation of siloxanes in a very short time. The optimization of chromatographic separation consisted in selecting the carrier gas flow rate and determining the temperature program of the column oven. Initial attempts to separate 3 siloxanes were conducted using a carrier gas flow of 1 mL/min, which was subsequently increased to 3 mL/min, to reduce the time required for the separation process of the analyzed compounds in up to 3 minutes. At the same time, the heating rate of the column was increased to 50°C/min. The skin extracts’ analysis confirmed the presence of large amounts of matrix components that appeared on the chromatograms mainly with longer retention times (Figure 3). It was necessary to increase the final working temperature of the column to 290 °C to allow high boiling point matrix components to leave the column. Under these conditions components of the skin matrix were effectively removed from the chromatographic system.

### 4.2. Validation of the Analytical Procedure and Acceptance Criteria

#### 4.2.1. Specificity

**Figure 2:**
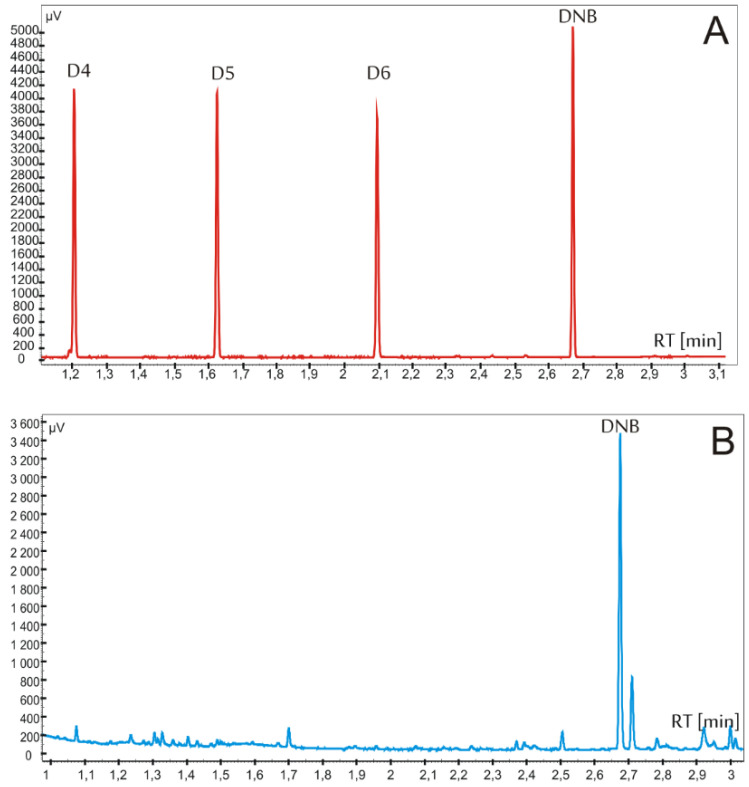
(**A**) separation of chromatographic peaks of the analyzed compounds D4, D5, and D6 and internal standard (DNB, 5 µg/mL); (**B**) example of matrix sample (stratum corneum).

**Figure 3:**
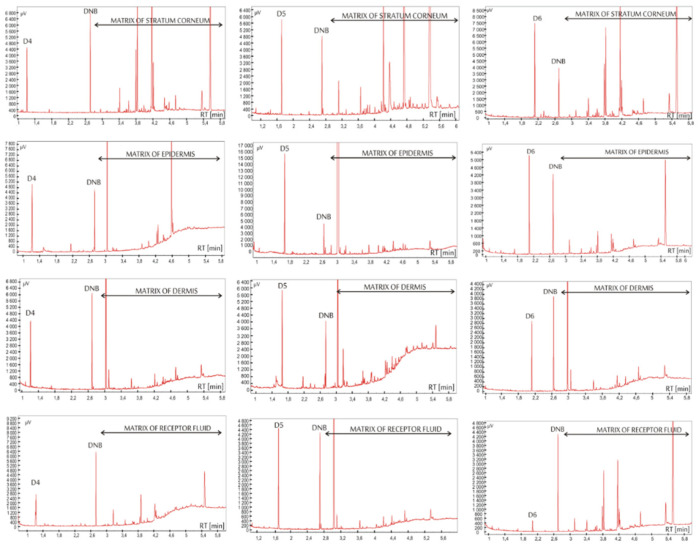
Representative chromatograms of the extracts obtained from the fractionated layers of the skin (stratum corneum, epidermis, and dermis) and from the receptor fluid containing octamethylcyclotetrasiloxane (D4), decamethylcyclopentasiloxane (D5), dodecamethylcyclohexasiloxane (D6), respectively; the internal standard (1,4-dinitrobenzene (DNB) was added at a concentration of 5 µg/mL.

The specificity was defined as the characteristic and reproducible retention time (*t*_r_) for the chromatographic peaks of analytes in the spiked and real samples. Due to the optimization process, the characteristic retention times for D4, D5, D6, and DNB (internal standard) peaks were as follows: 1.20, 1.63, 2.10, and 2.67 min, respectively (Figure 2). The results also confirmed that the dermal matrix or the receptor fluid did not interfere with the detection of siloxanes (Figure 2). The extracts using CCl_4_ as the solvent did not show the presence of any interfering contaminants. Figure 3 demonstrates representative chromatograms of the extracts containing D6. The chromatogram indicates an adequate separation of the target compounds.

#### 4.2.2. Precision and Repeatability of the Retention Times

In this study, we measured the retention time (*t*_r_) of three different concentrations of cyclic siloxanes: 1, 5, and 25 μg/mL, which constituted three measurements within three independent series of standard solutions. This allowed us to verify whether the amount of analyte entering the column would have an effect on the retention time. The impact of the matrix on the precision and repeatability of *t_r_* was also verified. The following results were obtained for the recovery test of D4, D5, and D6 added to CCl_4_ extracts of the human skin and receptor fluid Precision of *t*_r_ measurements for D4, D5, and D6 was 0.04–0.05%, 0.02–0.03%, and 0.02–0.04%, respectively. The recovery of D4, D5, and D6 was 0.04–0.16%, 0.04–0.11%, and 0.02–0.09%, respectively. The repeatability test for the standard solutions of D4, D5, and D6 was at the level of 0.04%, 0.02%, and 0.21%, respectively. Repeatability of the recovery test for D4, D5, and D6 was at a maximum of 0.10%, 0.08%, and 0.07%, respectively. No significant effect of the matrix was observed for the D4 retention time. Intermediate precision for the retention times was evaluated at the level of 3.88% for D4, 1.84% for D5, and 1.38% for D6. In the case of DNB present in standard solutions, the precision of *t*_r_ measurements was in the range of 0.01–0.03%, recovery was in the range of 0.03–0.31%, and the repeatability of the test was at a maximum of 0.02% and 0.13%. After several weeks, the intermediate precision was at the level of 0.88%.

#### 4.2.3. Linearity

For the quantitative analysis, we prepared a linearity curve with 10 different concentrations of standard solutions (range = 0.5–35 µg/mL). Figure 3 presents the relationship with the linear regression equation *y* = a*x* ± b of the calibration curve for compounds D4, D5, and D6. The regression equation was *y* = 0.1426*x* + 0.009 for D4, *y* = 0.1505*x* − 0.0141 for D5, and *y* = 0.1368*x* − 0.0091 for D6. The correlation coefficients of the standard curves were 0.9998, 0.9998, and 0.9995 for D4, D5, and D6, respectively. Based on the area ratio calculations, the precision and repeatability of measurements were determined. The precision was at the level of 0.8–5.8%, 0.7–4.6%, and 1.8–5.3% for D4, D5, and D6, respectively. The repeatability was calculated to be 2.8%, 1.9%, and 3.5% for D4, D5, and D6, respectively.

#### 4.2.4. Precision/Repeatability

The results for intra-day precision, which determines the precision of the method, and inter-day precision were satisfactory. The CV was in the range of ± 0.4–6.6% (Table 2). These data indicate that the assay method is reproducible within the same day and on different days.

#### 4.2.5. Accuracy/Trueness

Accuracy of cyclic siloxanes at 1, 5, and 25 μg/mL concentration was in the range of 99.7–106.1% (Table 2) and were acceptable.

#### 4.2.6. Sensitivity of the Assay, LOD, and LOQ

The calculated value of LOD and LOQ were sufficiently low to identify small amounts of siloxanes permeated into the different layers of the skin or in the receptor fluid. The LOD was 0.1 µg/mL, 0.2 µg/mL, and 0.1 µg/mL and LOQ was 0.3 µg/mL, 0.6 µg/mL, and 0.3 µg/mL for D4, D5, and D6, respectively. These values were low enough to guarantee the proper detection and quantification of cyclic siloxanes.

#### 4.2.7. Matrix Effect and Matrix Factor

In this study, the MF, which expresses the matrix effect, was verified by calculating the ratio between the response of the analyte including skin matrix sample at LQC, MQC, and HQC and the response of the analyte in the spiked solvent used for the extraction at LQC, MQC, and HQC (Section 3.2.6). Table 3 presents the calculated values of MFs for the investigation of the matrix effects for stratum corneum, epidermis, dermis, and receptor fluid. The skin matrix contains components that have an affinity to the solvent used, for example, ceramides, free fatty acids, and cholesterol [40]. Therefore, the purpose of the study was to demonstrate the possible impact of the extracted skin components on the signal of analytes. Our results have demonstrated that the matrix effect does not have a significant effect on the value of the concentration of cyclic siloxanes.

#### 4.2.8. Quality Control and Recovery (Extraction Efficiency)

Recovery pertains to the extraction efficiency of an analytical method within the limits of variability. Table 4 summarizes the results of siloxanes recovered from the stratum corneum, epidermis, dermis, and receptor fluid. The developed method demonstrated a high recoverability of cyclic siloxanes (>92.2%). The values obtained confirmed the expected recoveries according to the predictive equations established in the development of the extraction method. In the case of D4, the precision in the stratum corneum was in the range of 4.6–6.7%, with an accuracy of 95.2–105.5%; in the epidermis, the precision was in the range of 9.0–9.9%, with an accuracy of 100.1–102.6%; in the dermis, the precision was in the range of 4.3–5.2%, with an accuracy of 95.8–102.4%; in the receptor fluid, the precision was in the range of 2.8–4.6%, with an accuracy of 95.3–103.3%. In the case of D5, the precision in the stratum corneum was in the range of 5.9–7.3%, with an accuracy of 94.7–104.5%; in the epidermis, the precision was in the range of 5.7–11.3%, with an accuracy of 98.6–99.9%; in the dermis, the precision was in the range of 5.5–6.3%, with an accuracy of 93.9–103.4%; in the receptor fluid, the precision was in the range of 3.3–9.3%, with an accuracy of 98.7–101.0%. In the case of D6, the precision in the stratum corneum was in the range of 5.1–9.6%, with an accuracy of 92.2–101.6%; in the epidermis, precision was in the range of 5.8–9.2%, with an accuracy of 94.2–97.2%; in the dermis, precision was in the range of 5.7–6.6%, with an accuracy of 97.4–98.8%; in the receptor fluid, precision was in the range of 4.9–7.9%, with an accuracy of 97.6–103.0%. In all the above experiments, the CV was less than 15% [59]. Therefore, the recoveries indicate a very efficient extraction of the analytes and a high affinity of siloxanes to the solvent used (i.e., CCl_4_). Our results were consistent with the acceptance criteria for bioanalytical methods (Table 1).

Our results, with regard to the acceptance criteria were in accordance with the guidelines set forth by the FDA, EMA, and ICH for the validation of bioanalytical methods. Based on this, the validated GC-FID method was applied for the quantitative analysis of cyclic siloxanes (D4, D5, and D6) after extraction from the matrix. Furthermore, MF values were negligible; therefore, linearity, precision, and accuracy can be determined using standard solutions of siloxanes. This step is crucial, because skin samples are difficult to obtain due to ethical reasons.

### 4.3. Application of the Validated Method to Real Samples

The quantitative analysis demonstrated that human skin is not a barrier for cyclic siloxanes (silicones). Our results demonstrate the possibility of bioaccumulation and dermal absorption of these compounds. All the studied siloxanes were able to overcome skin barrier and permeate through the stratum corneum, epidermis to dermis and the receptor fluid. The data were shown as the cumulative doses of siloxanes (µg/cm^2^/24 h unit) in each layer of the skin, receptor fluid, and the total amount for each skin sample, as well as the concentration obtained after extraction (µg/mL) (Table 5). Our validated method provided us with repeatable and reliable results. It should be highlighted that a sample size of n = 7 is rarely found in this kind of bioanalytical research. It is noteworthy that in these types of experiments, it is possible to obtain large variations in the results (sometimes more than 15% or 20%) than what has been indicated in the guidelines for biological tests (Table 1). The variation in the results obtained for receptor fluid was nearly 30% (Table 5). This might be because of the specificity of the skin (which can vary depending on the site of the body or the origin from the different donors). Therefore, it is important to obtain the total amount (the sum of the cumulative doses for stratum corneum, epidermis, dermis, as well as receptor fluid).

**Figure 4:**
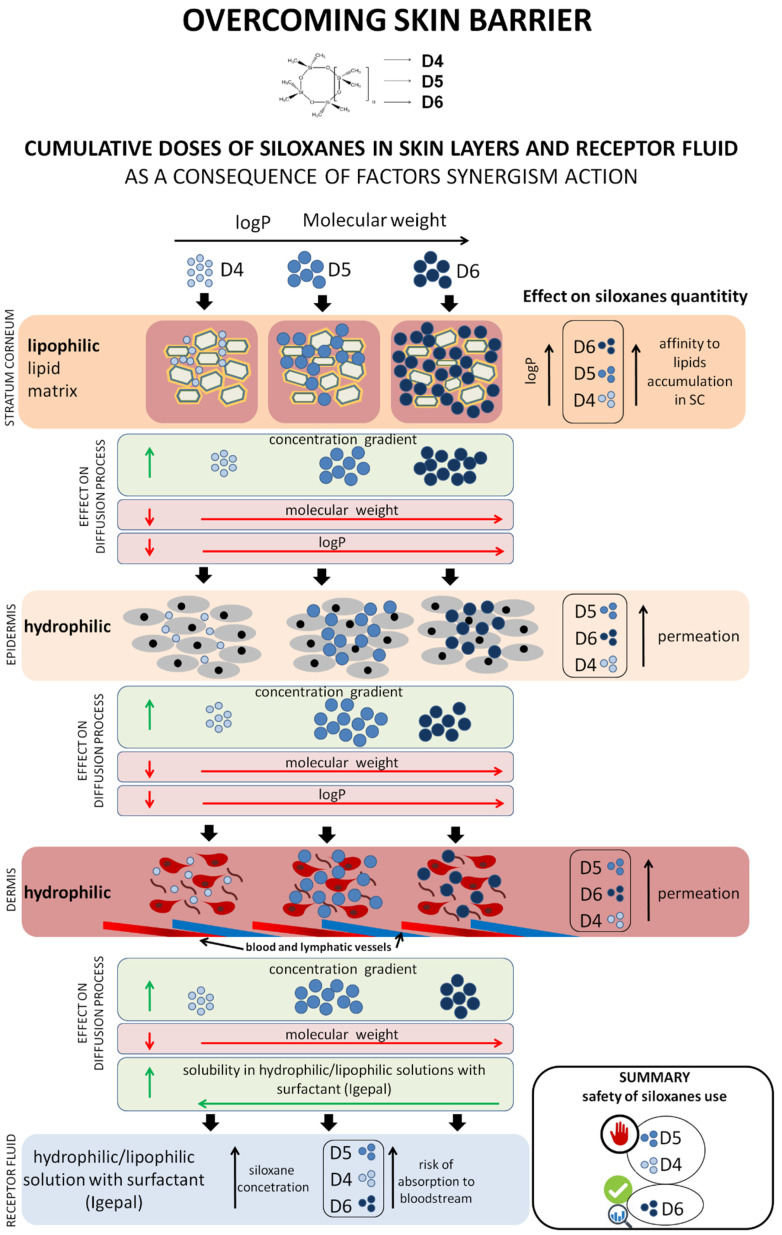
The mechanism of the overcoming human skin’s barrier system by various cyclic siloxanes.

According to the rule based on the Rule 5 by Lipinski, substances that are characterized by a molecular weight of <500 Da and lipophilicity (logP_o/w_) of lower than 5; however, more than 0.4 (preferably 1–3), number of proton donors HBD < 5; the number of proton acceptors HBA < 10 can more easily penetrate stratum corneum and permeate through the skin. Therefore, it is safe to assume that, because D4, D5, and D6 are characterized by a molecular weight of <500 Da, they were able to penetrate the stratum corneum and permeate into the deeper layers of the skin. However, in the case of siloxanes that have the logP_o/w_ value of greater than 8, there is a possibility of their accumulation in the lipid matrix, which will cause decreased diffusion process to deeper layers of the skin, resulting in the lower concentrations in receptor fluid. Taking the research hypotheses and our results into account, we were able to define the mechanism of how siloxanes overcome the skin’s barrier system. The amount of cyclic siloxane present in the individual of the skin is the result of the synergistic action of various factors (Figure 4). The most predominant factors that affect the process of diffusion are physicochemical properties of the test compounds (most importantly is their molecular weight and lipophilicity), solubility in the receptor fluid, specific skin structure, and components of skin layers, gradient concentration of these compounds between layers (stratum corneum, epidermis, and dermis), according to first Fick’s law, as well as the interaction between cyclic siloxanes and components of the stratum corneum (e.g., fats and proteins), which is described in detail by Krenczkowska et al. [7]. Thus, we can conclude that the mechanism of siloxanes in overcoming the skin’s barrier system was based on the same physicochemical laws and dependencies; however, their quantities differ in each layer of the skin and receptor fluid.

In the case of stratum corneum, the cumulative doses of D4, D5, and D6 were 27.5, 63.9, and 67.2 µg/cm^2^/24 h, respectively (Table 5).

Two essential phenomena determine the amount of siloxanes detected in the stratum corneum: diffusion and bioaccumulation. Bioaccumulation occurs in a highly lipophilic environment, which is surrounding the corneocyte cells. Our data shows that D4 can easily penetrate the stratum corneum; however, because it has the lowest value of lipophilicity, it did not accumulate in the layers of skin. It is noteworthy that bioaccumulation is related to the lipophilicity of the compound; larger molecules with a higher value of lipophilicity have a higher affinity to the lipophilic components of the stratum corneum which surrounds the corneocyte cells (e.g., ceramides (30–40%), fatty acids (approximately 18%), cholesterol and cholesterol sulfate (approximately 25%), and sterol esters (approximately 11%). D4 demonstrated the lowest value of the cumulative dose in the stratum corneum, whereas D6 was the most lipophilic of studied siloxanes. D6 was found to be accumulated in the horny layer after 24 h. Our results are comparable to those reported in a previous study, (Figure 4) [7].

In the case of the epidermis (Table 5), the highest cumulative doses were obtained for D5 (29.9 µg/cm^2^/24 h, lower amounts for D6 (10.7 µg/cm^2^/24 h), and the lowest for D4 (6.9 µg/cm^2^/24 h). Two different phenomena can be explained for the permeation of siloxanes from the stratum corneum into the epidermis: concentration gradient between the layers and chemical properties, including molecular weight and their lipophilicity. In the case of D6, despite the greatest concentration gradient of D6 between the stratum corneum and the epidermis, the permeated quantity was less compared to D5. In the epidermis, the cumulative amounts of D6 was 16% compared to that of stratum corneum, and the cumulative amounts of D4 and D5 in the epidermis was 25% and 47%, respectively. D6 is highly lipophilic, so it is difficult for it to permeate through the hydrophilic layer of the skin, i.e., epidermis. D4 demonstrated the smallest amount in the epidermis, which was principally caused by the lower concentration gradient formed between the layers of the skin.

In the case of the dermis, the cumulative doses of D4, D5, and D6 were 5.1, 13.9, and 5.3 µg/cm^2^/24 h (Table 5), respectively. D6 did not permeate to the hydrophilic dermis, because of its lipophilicity (logP = 8.87) and molecular weight (445 Da). D4 and D5 were able to permeate into the dermis more easily, because of their low molecular weight and high lipophilicity (297 and 371 Da, respectively) and logP (5.1 and 8.09, respectively). In the case of D4, the saturation extent of epidermis was smaller compared to D5 and in the consequence, the concentration gradient between epidermis and dermis was lower for D4 than for D5. About 74% of D4 and 46% of D5 had penetrated the dermis. In the case of D6, 50% of doses, in comparison to the cumulative dose in the epidermis (Table 5) were observed.

Our experiments revealed that the receptor fluids also contained D4, D5, and D6 (4.1, 7.4, and 0.9 µg/cm^2^/24 h, respectively) (Table 5). D6 was the lowest and D5 was detected in the highest cumulative doses. This might be because of the difference in the concentration gradient between the dermis and the receptor fluid. It was also claimed that the concentration gradient was not crucial in the case of a comparison of D4 and D6, due to the fact that the amounts of permeated siloxanes in the dermis are comparable (5.1 and 5.3 µg/cm^2^/24 h, respectively; Table 5). Therefore, in this case, the molecular weight, logP value, and solubility in hydrophilic/lipophilic receptor fluid should be considered while comparing their results. We analyzed the receptor fluid so that we could obtain an unsaturated state for all analytes (sink condition). Therefore, we added Igepal CA-630 at a concentration of 2% to the hydrophilic medium. The solubility of these compounds in water was also taken into account. The solubility of D4, D5, and D6 in H_2_O at 25 °C were 5.6, 5.1, and 1.7 µg/L, respectively. In summary, 80% of D4, 53% of D5, and 17% of D6 were detected in receptor fluid in comparison to the cumulative dose in dermis (Table 5). D4 was found to permeate more easily into the receptor fluid compared to D6.

In summary, it should be highlighted that the amount of D4, D5, and D6 applied to the skin was practically equal; the volume applied was 100 µl, which corresponded to about 95,600 mg (chapter 2.6) if density (0.950–0.963 g/cm^3^) was taken into account.

The analysis of permeation from stratum corneum to epidermis revealed the fact that the studied siloxanes differed in their affinity to stratum corneum. The cumulative doses of D4, D5, and D6 found in epidermis were 6.9, 29.9, and 10.7 µg/cm^2^/24 h, respectively, which confirmed the highest affinity of D6 to stratum corneum as the amount diffused into the epidermis was 2.8 times smaller compared to D5. This may also indicate that the affinity to stratum corneum is not the only determinant in this case. The process of permeation into epidermis is more complex, because it is also determined by the differences in the affinities of D5 and D6 for this layer. The physicochemical properties of epidermis cause higher inhibition of the diffusion of D6 compared to D5, as D6 is more lipophilic than D5. From the understanding of the impact of various factors on the complexity of the diffusion process, it can be concluded that the similarity of D5 and D6 cumulative doses in stratum corneum (63.9 and 67.2 µg/cm^2^/24 h, respectively) results from the saturation state of the two compounds in this layer.

The calculated epidermis-to-stratum corneum ratios of analyte concentrations (Table 5) confirm the above reasoning. The largest ratio was identified for D5 (E/SC = 47), followed by D4 (E/SC = 25), and finally by D6 (E/SC = 16).

The analysis of the next stage of diffusion from epidermis to dermis revealed that in dermis the highest cumulative dose was observed for D5 (13.9 µg/cm^2^/24 h), while the doses of D4 and D6 were similar (5.1 and 5.3 µg/cm^2^/24 h, respectively). Considering the concentration gradient, it can be concluded that the diffusion of D5 and D6 occurs at a similar level, while D4 diffuses at a much higher level. These observations were also confirmed by the dermis-to-epidermis concentration ratios calculated for the analytes (D4: D/E = 74, D5: D/E = 46, and D6: D/E = 50) (Table 5).

The final stage of diffusion from dermis to the receptor fluid indicated that D4 was able to permeate easily, while D5 exhibited a difficult diffusion and the diffusion of D6 was limited. Although the cumulative doses of D4, D5, and D6 in the receptor fluid were 4.1, 7.4, and 0.9 µg/cm^2^/24 h, the receptor fluid-to-dermis concentration ratios (RF/D) were calculated to be 80, 53, and 17, respectively.

Another important observation was the differences in the ratios of analyte concentrations in individual matrices compared to stratum corneum. The stratum corneum layer played a crucial role, forming the initial concentration gradient. The calculations indicated the following:

(a) D5 had a greater affinity for stratum corneum and epidermis, compared to D6.
(b) The greater concentration gradient, compared to D4, was the driving force for the diffusion of D5 from stratum corneum to epidermis. The epidermis-to-stratum corneum ratios were—D4: E/SC = 25, D5: E/SC = 37, and D6: E/SC = 16.
(c) The diffusion of D4 and D5 from epidermis to dermis, and subsequently from dermis to the receptor fluid, was similar, which may indicate their similar affinity for the hydrophilic epidermis and dermis layers, as well as solubility in the receptor fluid. The calculated ratios for D4 were D/SC = 19 and RF/SC = 15, and that for D5 were D/SC = 22 and RF/SC = 12. Definite differences were observed for D6, for which the ratios were calculated as D/SC = 8 and RF/SC = 1. This indicates a significantly diminished diffusion process, despite the fact that the study was conducted in sink conditions.

Our study proved the hypothesis that ex vivo human skin is not a barrier for cyclic siloxanes, which are frequently used in the preparation of dermatological, cosmetic, and personal care products. The application of these compounds on the skin’s surface contributes to their penetration into the stratum corneum layer, permeation to the deeper layers of the skin (epidermis and dermis), and finally into the receptor fluid. These compounds can eventually enter the bloodstream. According to our results, D6 showed the lowest risk of absorption compared to D4 and D5. Apart from this, it is important to note that cyclic siloxanes can bioaccumulate in the dermis.

## 5. Conclusions

The validated quantitative GC-FID analysis demonstrated that ex vivo human skin is not a barrier to cyclic siloxanes. D4, D5, and D6 have a specific affinity to stratum corneum (especially D6), and can even diffuse into the deeper layers of the skin (epidermis and dermis) or into the receptor fluid as well. An important achievement of the indicated works was their observation that the complexity of skin tissue and barriers as well as the differentiation of physical–chemical properties of cyclic siloxanes resulted in their ratio partitioning in skin layers and receptor fluid. Consequently, the correlation between features was established, which explained the realistic mechanism behind the diffusion of these compounds into the skin. The studies have shown that, in order to thoroughly understand the mechanism, it is important to determine not only the differences in the amounts of cumulated doses [µg/cm^2^/24 h], but also the mutual ratios of analyte concentrations existing in each skin layer (epidermis-to-stratum corneum ratio and dermis-to-epidermis ratio), as well as in the receptor fluid (receptor fluid-to-dermis ratio).

The ex vivo studies presented in this paper imply that the real threat should be considered a result of the daily use of dermatological and cosmetic products containing D4, D5, and D6. Thus far, exposure has been estimated only by determining the content of cyclic siloxanes in the skin products or the concentrations in serum after a cosmetic or body care product was applied [32,34,60]. However, data obtained from serum demonstrate only the concentration of siloxanes present at the time of analysis, and do not reveal the possible extent of accumulation and subsequent long-term release from lipophilic reservoir, such as skin layers—stratum corneum or subcutaneous tissue. The process of stratum corneum desquamation takes about 14 days; therefore, the doses accumulated in stratum corneum can possibly increase after the daily use of a product. This phenomenon can lead to the diffusion of cyclic siloxanes into the deeper skin layers, or even the lymphatic and blood vessels, with long-term use. The dermis can also considered a reservoir. Bioaccumulation in the dermis can impact on release at a later time, which means that it will be the potential dose which can be absorbed to the bloodstream in a long-term and uncontrolled process.

Although the knowledge acquired in the present work can be valuable for the subsequent risk evaluation of the usage of dermal products containing cyclic siloxanes of low molecular weight, it should be emphasized that, in this study, an infinite dose of each cyclic siloxane was applied on the top of the skin, while, for testing the dissolution of cyclic siloxanes from the skin products, a finite dose was used in a concentration appropriate for the product type (e.g., ointment, lotion, emulsion, cream). The use of an infinite dose not only enabled identifying the realistic mechanism behind the penetration and permeation of the studied siloxanes, but is used when no such research has been carried out so far. We would also like to point out that according to the guidelines of OECD and WHO, ex vivo human skin studies are the gold standard for transdermal research, and can demonstrate the closest relationship with the in vivo processes [61].

## Figures and Tables

**Table 1:** Selected recommendations and acceptance criteria for the bioanalytical and analytical methods in accordance with the United States Food and Drug Administration (FDA), European Medicines Agency (EMA), and International Conference on Harmonization (ICH) guidelines [53,54,55,56,57,58].

Studies	Validation Parameter	Acceptance Criterion
**Qualitative**	Specificity	Blank samples of the appropriate biological matrix should be obtained from at least six individual sources. The response of the interfering compound should be less than 20% of the lowest concentration of the standard curve lower limit of quantification (LLOQ) and 5% standard response internal standard (IS). Blank and zero calibrators should be free of interference at the retention times of the analyte(s) and the IS. Spiked samples should be ± 20% LLOQ. The IS response in the blank should not exceed 5% of the average IS responses of the calibrators and quality controls (QCs).
	Precision/repeatability/intermediate precision for retention time	Lack of the acceptance criterion in guidelines. Adopted ≤ 2.5%.
**Quantitative**	Range	Analogically to linearity
	Linearity	The lowest concentration of analyte from the calibration curve LLOQ ≥ 5 times higher than the blank sample, with a precision of up to 20% of the coefficient of variation (CV) and accuracy within ± 20% (80–120%); upper limit, the highest concentration of the standard curve upper limit of quantification (ULOQ) with a precision below 15% CV and accuracy within ± 15% (85–115%); the correlation coefficient (r) should be greater than 0.99 and the coefficient of determination be (r^2^) 0.98. Nonzero calibrators should be ± 15% of the nominal (theoretical) concentrations (NC), except at LLOQ where the calibrator should be ± 20% of the nominal concentrations in each validation run. In addition, 75% and a minimum of six nonzero calibrator levels should meet the above criteria in each validation run.
	Sensitivity	The lowest nonzero standard on the calibration curve defines the sensitivity (LLOQ). The analyte response at the LLOQ should be ≥ 5 times the analyte response of the zero calibrator. The accuracy should be ± 20% of the nominal concentration (from ≥ 5 replicates in at least 3 runs). The precision should be ± 20% of the CV (from ≥ 5 replicates in at least 3 runs).
	LOQ	A 10:1 signal-to-noise ratio. The quantitation limit is generally determined by the analysis of samples with known concentrations of analyte and by establishing the minimum level at which the analyte can be quantified with an acceptable accuracy and precision.
	LOD	A 3:1 signal-to-noise ratio.
	Precision/Repeatability	Precision should be established with at least 3 independent runs, 4 QC levels per run (LLOQ, L, M, and H QC), and ≥ 5 replicates per QC level. CV in the biological matrix should not exceed 15% and close to 20% of the LLOQ value.
	Accuracy/Trueness	Accuracy should be established with at least 3 independent runs, 4 QC levels per run (LLOQ, L, M, and H QC), and ≥ 5 replicates per QC level. The relative error should not exceed ± 15% of the actual value, except for the lowest concentration of the LLOQ analyte close to ± 20% of the value.
	QCs	Four QCs, including LLOQ, low (L: defined as three times the LLOQ), mid (M: defined as mid-range), and high (H: defined as high-range) from at least 5 replicates in at least 3 runs. The QCs should be prepared from separate stock solutions than the calibration standards in order to avoid biased estimations that are not related to the analytical performance of the method.
	Recovery (extraction efficiency)	Extracted samples at low quality control (LOQ), medium quality control (MOQ), high quality control (HQC) concentrations versus extracts of blanks spiked with the analyte postextraction (maximum of 3 times the LLOQ and close to the HLOQ). Analyte recovery does not have to be 100%, but the analyte and IS recovery rates should be precise and reproducible. The extraction efficiency of an analytical process, reported as a percentage of the known amount of an analyte carried through the sample extraction and processing steps of the method (sample pre-extraction versus sample postextraction).
	Matrix effect	Extracted samples including matrix at L, M, and H QC concentrations versus spiked solvent at L, M, and H QC concentrations used for the extraction. It should be assessed by analyzing at least 6 lots of matrix, spiked at a low and at a high level of concentration (maximum of 3 times the LLOQ and close to the ULOQ). The calculated matrix factor (MF) should be 1. The overall CV calculated for the concentration should not be greater than 15%.

**Table 2:** Validation results (intra-day precision, inter-day precision, and accuracy) of the analytical method for the determination of D4, D5, and D6 (n = 6/18).

Cyclic Siloxane	Nominal Concertation NC [µg/mL]		Concentration Found CF (Mean) [µg/mL]	Intra-Day Precision CV [%] n = 6	Inter-Day Precision CV [%] n = 18	Accuracy [%] n = 6
D4	LQC	1	1.0	2.4	3.6	102.8
	MQC	5	5.0	1.5	2.2	100.4
	HQC	25	24.8	0.6	1.1	99.3
D5	LQC	1	1.0	6.6	4.7	106.1
	MQC	5	5.1	1.7	1.9	100.44
	HQC	25	25.3	0.4	1.1	100.7
D6	LQC	1	1.0	2.0	6.2	105.7
	MQC	5	5.1	3.5	2.9	105.6
	HQC	25	24.2	6.5	5.7	99.7

**Table 3:** Matrix effect coefficient for D4, D5, and D6.

Siloxane	Heading	Matrix Effect			
	Nominal Concentration [µg/mL]	Stratum Corneum	Epidermis	Dermis	Receptor Fluid
D4	1	1.03	1.01	0.99	0.93
	5	0.92	1.07	0.95	1.03
	25	0.98	1.03	1.10	1.04
D5	1	0.98	0.93	0.97	0.93
	5	0.94	0.98	0.93	0.98
	25	0.94	0.97	0.99	0.99
D6	1	0.96	0.91	0.92	0.97
	5	0.89	0.91	0.94	0.94
	25	0.94	0.96	0.97	1.07

**Table 4:** Results of quality control and recovery (extraction efficiency) of D4, D5, and D6 from the stratum corneum, epidermis, dermis, and receptor fluid, n = 6.

Cyclic Siloxane	Heading	Sample	Stratum Corneum			Epidermis			Dermis			Receptor Fluid		
		NC [µg/mL]	CF [µg/mL]	CV	Accuracy	CF [µg/mL]	CV	Accuracy	CF [µg/mL]	CV	Accuracy	CF [µg/mL]	CV	Accuracy
				(%)	(%)		(%)	(%)		(%)	(%)		(%)	(%)
D4	LQC	1	1.1	6.7	105.5	1.0	9.0	102.6	1.0	4.6	100.8	0.9	2.8	95.3
	MQC	5	4.8	4.6	95.2	5.0	9.3	100.1	4.8	4.3	95.8	5.2	4.6	103.3
	HQC	25	24.3	5.3	97.1	25.4	9.9	101.6	25.5	5.2	102.4	25.6	3.5	102.2
D5	LQC	1	1.0	6.8	104.5	1.0	5.7	99.9	1.0	5.8	103.4	0.9	3.3	99.5
	MQC	5	4.7	7.3	94.7	5,0	10.6	99.2	4.7	5.5	93.9	4.9	5.3	98.7
	HQC	25	23.7	5.9	94.9	24.7	11.3	98.6	25.1	6.3	100.5	25.3	9.3	101.0
D6	LQC	1	1.0	5.1	101.6	1,0	5.8	97.2	1.0	5.7	98.1	1.0	4.9	103.0
	MQC	5	4.6	9.6	92.2	4.7	7.4	94.2	4.9	6.6	97.4	4.9	7.9	97.6
	HQC	25	24.1	7.4	96.2	25.6	9.2	97.4	24.7	5.9	98.8	25.7	6.5	102.7

**Table 5:** The cumulative doses (CD) of D4, D5, and D6 in various layers of the skin and in receptor fluid; total amount after diffusion as well as concentration in extracts (CE); calculated epidermis-to-stratum corneum, dermis-to-epidermis, dermis-to-receptor fluid ratios. mean obtained from n = 7.

Sample	D4				D5				D6			
	CD [µg/cm^2^/24 h]	CE [µg/mL]	CV [%]	Ratio [%]	CD [µg/cm^2^/24 h]	CE [µg/mL]	CV [%]	Ratio [%]	CD [µg/cm^2^/24 h]	CE [µg/mL]	CV [%]	Ratio [%]
Stratum corneum	27.5	3.5	4.9	25	63.9	8.1	5.5	47	67.2	8.6	20.3	16
Epidermis	6.9	4.4	21.4		29.9	19.1	9.4		10.7	6.8	6.2	
				74				46				50
Dermis	5.1	3.2	24.5		13.9	8.8	13.5		5.3	3.4	7.4	
				80				53				17
Receptor fluid	4.1	2.6	27.4		7.4	4.7	24.2		0.9	0.6	29.5	
TOTAL	43.6	13.7	8.8		114.1	40.7	2.6		84.1	19.4	16.3	

## Abbreviations

GC-FID	gas chromatography-flame ionization detector
SC	stratum corneum
TS	tape-stripping
FDA	United States Food and Drug Administration
EMA	European Medical Association
ICH	International Conference on Harmonization
CV	coefficient of variation
IS	internal standard
MF	matrix factor
QCs	quality controls
LOQ	limit of quantification
LOD	limit of detection
LLOQ	lower limit of quantification
ULOQ	upper limit of quantification
DNB	1,4-dinitrobenzene
D4	octamethylcyclotetrasiloxane
D5	decamethylcyclopentasiloxane
D6	dodecamethylcyclohexasiloxane
LQC	low quality control
MQC	medium quality control
HQC	high quality control
NC	nominal concentration
CF	concentration found
CD	cumulative dose
CE	concentration in the extract
